# Between policy and pedagogy: writing teachers' perspectives on institutional AI policies and expectations

**DOI:** 10.3389/frai.2026.1926666

**Published:** 2026-10-09

**Authors:** Mohammed Alzahrani, Faris Aldubayyan, Ibrahim Alshabi

**Affiliations:** English Language Department, College of Science and Health Professions, King Saud bin Abdulaziz University for Health Sciences, King Abdullah International Medical Research Center, Ministry of National Guard/Health Affairs, Riyadh, Saudi Arabia

**Keywords:** AI policies, educational policy, policy enactment, teacher perceptions, writing teachers

## Abstract

Despite the growing interest and rapid advancements in artificial intelligence (AI) within higher education, English language teachers remain in a constant state of interpreting, implementing, and negotiating institutional policies and expectations. While rapidly developed AI policies have started to emerge and evolve, little is known about how writing teachers understand, evaluate, and negotiate these policies, especially in relation to their everyday pedagogical practices. This qualitative study is set to explore how 10 writing teachers viewed and navigated their institutional AI policies in Saudi Arabia. Utilizing a case-study design, data were collected from narrative frames and semi-structured interviews. The results revealed that the teachers expressed a lack of clear institutional guidance, leading them at times to navigate AI-related issues on their own. Additionally, the teachers underlined their partial exclusion from AI policy design, which mainly followed a top-down institutional approach. Despite that, teachers perceived AI adoption as an inevitable development within higher education and a necessity that they need to adapt to in the future. The study underscores the importance of designing clear AI policies, tailored specifically to help writing teachers working in higher education settings. It also highlights how a bottom-up approach, where teachers are involved in the policy-drafting and implementation phases, could have a fundamental and constructive effect on their pedagogical practices.

## 1 Introduction

With the advancements of technology, artificial intelligence (AI) is increasingly being recognized and adopted across a wide range of settings, including higher education (Ghimire and Edwards, 2024). In writing contexts, assumptions regarding authorship and assessment have been debated and contested by many scholars over the past few years (Al-Othman, 2024; Cotton et al., 2024; Kasneci et al., 2023). These debates led to the emergence of institutional policies, whose goal was to help make clear what constitutes the appropriate use of AI and what does not. Although establishing such policies has its merits, these policies do not fully account for the pedagogical realities experienced by writing teachers, resulting in a gap between policy formulation and classroom practice. One limitation that arises with regard to previous studies lies in the tendency to view AI in a binary way, portraying it as either a threat to academic integrity (e.g., Cotton et al., 2024) or as a pedagogical opportunity (e.g., Kasneci et al., 2023). Such binary positions unintentionally help obscure the complex and situated judgments teachers must make when navigating institutional demands. Moreover, institutional AI policies have been portrayed to have a top-down approach that could marginalize teacher agency and professional expertise (Tsao, 2025). This study contributes to these debates by attaining writing teachers' views on institutional AI policies and expectations, which is an area that remains underexplored despite its crucialness to effective policy enactment. In the context of this study, institutional AI policies refer to formally articulated institutional rules or guidelines regarding the use of AI in teaching, learning, and assessment. On the other hand, Informal expectations, departmental discussions, and other forms of guidance are treated as institutional expectations rather than formal institutional policies. Current policy discourse often fails to account for teachers' interpretive labor, instead assuming equal and uniform implementation across diverse teaching contexts. Such assumptions are particularly problematic in writing classrooms, where originality, voice, and process are considered already contested (Matsuda, 2015; Pennycook, 1996). By capturing writing teachers' views, this study explores how they navigate institutional AI policies and/or expectations. It challenges the notion that positions policy as either enabling or constraining and, instead, brings forward the nuanced and often contradictory ways teachers engage with AI governance. In doing so, the study contributes to the scholarly discussion regarding the need for more context-sensitive policy frameworks that take into account teachers as key stakeholders in shaping the future of writing in the age of AI.

## 2 Literature review

### 2.1 AI and writing in higher education

The growing expansion of AI has extended scholarly discussion regarding its pedagogical role in higher education writing contexts. Rather than merely functioning as a supplementary tool, contemporary AI systems, specifically large language models, are increasingly perceived as active participants in academic writing and research venues (Tang, 2025). Specifically,

“AI utilizes machine learning, neural networks, and other techniques to generate new content (e.g., text, images, and music) by analyzing patterns and information from the training data” (Ooi et al., 2025, p. 76).

In contrast to traditional AI platforms, which typically operate through predefined rules for pattern recognition and data classification, AI can generate novel outputs across formats such as text, images, and other media that were neither explicitly programmed nor previously observed (Dwivedi et al., 2023). What these advancements entail is their ability to create sophisticated human-like content (Lim et al., 2023). Existing research, however, describes this development as being greatly ambivalent, where it simultaneously documents measurable benefits while raising pedagogical concerns (Dwivedi et al., 2023; Kasneci et al., 2023).

AI-powered writing tools, for example, have shown increasingly reported benefits in supporting students' overall writing development. Aljuaid (2024) argues that AI applications such as Grammarly, QuillBot, and ChatGPT can, in fact, improve grammatical accuracy as well as the overall writing quality while simultaneously increasing students' confidence and productivity during the writing process. These tools operate through natural language processing trained on large textual corpora, which enables them to generate paraphrased texts while maintaining linguistic sophistication. This could result in students' perceptions of these tools as accessible writing assistants that reduce cognitive load during their writing. Moreover, contemporary AI writing platforms can extend beyond minor editing, as they could support and help with both summarization and the generation of sophisticated draft feedback (Khalifa and Albadawy, 2024). Such support could help English learners focus more on content development rather than linguistic form. When viewed this way, AI tools allow for a more diverse participation in academic writing by lowering language barriers.

Nevertheless, despite such benefits, research constantly warns against its uncritical adoption. A recurring point of argument is the possible decline in students' critical thinking and intellectual independence. Aljuaid (2024) states that although AI can assist with grammar and style, academic writing courses help develop much more complex competencies, such as argumentation and critical reasoning, which current AI systems are still unable to fully replicate. This distinction is rather important, since it positions AI as supportive rather than as substitutive. Fan et al. (2025) also warn that excessive reliance on AI may lead to shallow learning if students neglect the cognitive effort required for basic knowledge construction. Their analysis suggests that AI can lead to cognitive laziness, which is further fostered when learners depend on ready-made AI outputs. This is how AI use can introduce a pedagogical dilemma between efficiency and meaningful learning.

More importantly, given that AI systems are becoming more normalized among students in their writing practices, higher education institutions are compelled to take action to account for academic integrity while also acknowledging the growing inevitability of AI-assisted writing. This made universities worldwide move toward constructing policies and guidelines designed to regulate the appropriate role of AI in teaching, learning, and assessment (Jin et al., 2025; Moorhouse et al., 2023). However, it is crucial to mention that the effectiveness of these institutional responses remains uncertain, as little is known about how faculty members interpret or contribute to the formation of institutional AI governance frameworks.

### 2.2 Institutional AI policies and faculty perceptions

Scholarly and higher education institutions have begun to move rapidly to establish policies and guidelines regarding the use of AI (An et al., 2025; McDonald et al., 2025). Formal policies are more than mere procedural documents and are rather representative of institutional attempts to negotiate the pedagogical and ethical uncertainties that arise from AI-mediated writing. At the institutional level, early responses regarding the use of AI were often fragmented and provisional. Spivakovsky et al. (2023) stated that many universities initially lacked coherent regulatory guidelines and instead relied entirely on individual teachers' judgment. Understandably, this approach resulted in inconsistent guidance and, in some cases, uncertainty among both faculty and students regarding the appropriate use of AI (Cotton et al., 2024; Maistry, 2025). Similar patterns were also observed globally following the public release of ChatGPT, when many institutions found themselves in a

“state of organizational paralysis, adopting a ‘wait and see' strategy” (Korseberg and Elken, 2025, p. 953).

Shortly after, research started shifting toward recognizing how institutions are gradually moving toward more systematic policy guidelines. Jin et al. (2025) found out that institutions are now emphasizing academic integrity and ethical AI use as core priorities of their institutional policies. This resulted in a shift away from prohibition-oriented perspectives toward more adaptive frameworks that combine guidance, training, and assessment design (Cotton et al., 2024; Zhai, 2023). This move is reflective of the growing recognition that AI is becoming an embedded and active element in the structures of academic work.

When shifting the scope to how faculty interact with AI policies, studies showed that the effectiveness of these policies depends less on their formal content and more on how they are interpreted and implemented by faculty under conditions of uncertainty (Cotton et al., 2024; Zhai, 2023). This uncertainty leads to teachers having to navigate AI without clear or stable guidance, which consequently results in mixed and often cautious responses (Bearman et al., 2023; Farrokhnia et al., 2024). This was particularly investigated in a faculty survey administered by Petricini et al. (2024). They concluded that despite the fact that faculty were familiar with AI and viewed its use as inevitable, they remained uncertain about appropriate applications and institutional expectations. Mamo et al. (2024) likewise advocated in favor of this notion. They argued that faculty attitudes toward AI tend to be ambivalent, as they show interest in its pedagogical potential but simultaneously express concerns about its misuse and reliability. It is also important to mention that when AI implementation occurs without clear policies or faculty involvement, teachers often experience ambiguity as well as a reduced sense of autonomy (Buele and Llerena-Aguirre, 2025). These findings bring to light that institutional AI governance is often handled locally through faculty judgment rather than being uniformly and structurally applied.

Having said that, the trajectory of institutional policy suggests movement toward regulated and structured integration rather than prohibition or unrestricted adoption (Cotton et al., 2024; Kasneci et al., 2023; Korseberg and Elken, 2025). Universities and institutions appear to be establishing adaptive models that acknowledge the growing inevitability of AI-assisted writing while attempting to account for academic values such as academic integrity and authorship accountability.

While previous research has navigated the accelerated development of institutional AI policies (Korseberg and Elken, 2025; Cotton et al., 2024; Farrokhnia et al., 2024), the implementation of such policies remains uneven and inadequate. In many contexts, including Saudi Arabian higher education, guidance regarding AI and proper implementation of AI policies are often absent, which could lead to teachers navigating AI ambiguously and uncertainly. As a result, it remains less understood how teachers navigate institutional policies and expectations toward AI when formal written policies are absent or unclear. This gap is especially consequential in writing instruction, where questions of authorship and originality are inherently fundamental. Existing studies mainly focused on institutional documents or the general faculty attitudes toward AI, with limited attention to discipline-specific contexts (Bearman et al., 2023; Cotton et al., 2024; Korseberg and Elken, 2025; Tlili et al., 2023). To address this gap, the present study examines writing teachers' perspectives and practices in relation to institutional AI policies and expectations. Specifically, it addresses the following overarching research question: How do writing teachers in a Saudi university view and navigate institutional AI policies and/or expectations?

## 3 Methodology

### 3.1 Context and participants

The context of the current study is a preparatory program at a Saudi university in which English is the medium of instruction. The program is designed to serve students in their first 2 years and prepare them for their following studies at their respective colleges. In doing so, students are enrolled in 7 English language courses in their first 2 years. Other courses include humanities courses delivered in Arabic and science courses primarily conducted in English. The participants in this study were English writing teachers who have been involved in teaching such students. They come from various backgrounds, hold different English teaching degrees, and have considerable teaching experience in the program (Table 1). Such teachers are generally involved in teaching three writing courses across three semesters in the program. Purposive sampling was chosen to recruit participants who had lengthy experiences in teaching writing. Notably, those teachers have been part of the program before and after the advent of AI. Accordingly, eligible participants were invited in person, and participation was voluntary. The research study was approved by the IRB office at the researchers' institution, and all ethical considerations were followed, ensuring the anonymity of personal data and participants' information.

**Table 1:** Participants' demographic information.

Teacher	Teaching experience in the program	Degree	Background
T1	12	BA	American
T2	12	MA	British
T3	12	BA	British
T4	13	Ph.D.	British
T5	11	MA	Australian
T6	12	MA	American
T7	12	MA	British
T8	12	Ph.D.	American
T9	12	MA	British
T10	11	MA	British Canadian

### 3.2 Research design and data collection

The study utilized a qualitative design for data collection and analysis. Specifically, a case study design was chosen to explore how writing teachers at a Saudi university perceived and navigated their institution's views on AI policies or lack thereof. In order to explore this area, the choice of a case study design was set to offer an in-depth description of the teachers' lived experiences of the phenomenon. Of particular interest is how a case study could capture the complexities of a research problem, acknowledging at the same time that the main purpose for such a choice of design is not mainly to generalize results, but rather to offer a particularistic and context-bound view of a specific case (Stake, 1995). Additionally, the study follows Yin (2018) description of a case study in the sense that (1) the research question is set to answer *how* a phenomenon is experienced, (2) there is no control over behavioral outcomes, and (3) the focus of the study under scrutiny is contemporary.

In the first stage of data collection, data were collected through the completion of written narrative frames, which are defined by Barkhuizen (2011) as:

“*A written story template consisting of a series of incomplete sentences and blank spaces of varying lengths. It is structured as a story in skeletal form. The aim is for participants to produce a coherent story by filling in the spaces according to their own experiences and their reflections on these*” (p. 402).

The inclusion of narrative frames was important, especially at the early stages of data collection, as the teachers' narratives were meant to

“ensure that the content will be more or less what is expected (and required to address the research aims) and that it will be delivered in narrative form” (Barkhuizen and Wette, 2008, p. 376).

Accordingly, two narrative frames were designed, following Barkhuizen (2014) design features, to capture the teachers' standpoints with regards to their institutional expectations toward AI implementation. These frames were developed based on the study's research questions and the relevant literature on institutional AI policies and were designed and reviewed by the first two researchers. They consisted of multiple incomplete sentences designed for participants to narrate their perceptions of institutional AI policies. Specifically, the first narrative frame focused on teachers' overall experience with AI. This is evident in the following brief example:

Over the years of my teaching experience, I have encountered the implementation of some technologies in teaching and learning, but the advent of AI was………… I first heard about AI in/through………… I remember that I felt………… I thought AI would………… because…………

The second narrative frame addressed more explicitly any institutional policies and expectations toward AI usage, and how the teachers responded to them. An example from this frame is the following excerpt:

The first time AI integration was brought into discussion in my institution was when………… During that time, the institution's expectations on AI were………… Initially, I felt ………… because………… However, these expectations are now………… The current institutional policies on AI are………… These policies influence my teaching through…………

It is also important to mention that the first narrative frame was completed electronically by the participants prior to conducting the interviews, and the second one was similarly filled after the interviews to allow the participants to reflect on what they had already expressed in the first frame.

Semi-structured interviews were also developed to get a holistic and detailed picture of the participants' accounts of institutional AI policies. They were conducted individually with the teachers to get a holistic picture of their narratives and views on their institution's standpoint on AI. We specifically followed Creswell (2013) detailed approach in both the design and implementation of interviews, allowing us to delve deeper into teachers' understandings of AI and the meanings they assigned to their institution's expectations and policies. The interview protocol consisted of open-ended questions that revolved around four areas: (1) teachers' current experiences of AI integration, (2) the strategies they used to regulate AI usage, (3) teachers' stories about and responses to students' use of AI, and (4) their institution's policies and expectations toward adopting AI in teaching and learning. Follow-up questions were used to support elaboration and probe more details, stories, or issues raised by the participants. Most importantly, the interview questions were designed based on the study's research question and was used to complement rather than duplicate data gained from the narrative frames. Additionally, each interview lasted for approximately 30–40 mins, was conducted in English, and was accordingly transcribed and prepared for analysis. The data collection period took over the course of one academic semester. The interviews were conducted individually by either the first or the second researcher.

Overall, each teacher completed the first narrative frames and was subsequently invited to conduct the interviews and the second narrative frame. The first narrative frame served to initially elicit participant's experiences, while the second frame and interviews were particularly meant to provide an opportunity to contextualize participants' perceptions as well as expand on any views mentioned initially in the first narrative frame. It should also be noted that this case study is situated within one Saudi university. Based on that, the study is bounded by the teachers' specific experiences and perceptions of institutional AI policies within this university. Therefore, the aim of this study is not to generalize its results to other contexts but rather assess the transferability of its main findings to other similar contexts (Lincoln and Guba, 1985).

### 3.3 Data analysis

Both sources of data were analyzed separately and compared during the coding and theme development stages. The use of narrative frames and interviews allowed us to connect and move between the specific accounts mentioned in the first narrative frame and the detailed descriptions expressed later in the interviews and the second narrative frame. Specifically, to analyze the data, we followed Braun and Clarke (2006) six-stage method for thematic analysis. First, the first two researchers started familiarizing themselves with data by listening to the interviews multiple times, followed by repeated readings of the narrative frames and transcribed interviews. Next, the two researchers met to discuss the initial codes that were identified across all data sets. After agreeing on a set of codes, initial themes were developed by the first two researchers and discussed in three separate meetings that included all researchers. During this stage, the three researchers assessed whether the codes adequately captured the developed themes. Following that, themes were consequently reviewed and named by all researchers. Any disagreements regarding coding or thematic development within these stages were resolved through iterative discussions and refinement of the themes until consensus was reached. In doing so, we ensured that continuous comparison across the teachers' perspectives was made to allow for coherence and representativeness. This recursive process allowed us to move between individual accounts and larger thematic patterns within the dataset.

To enhance the trustworthiness of the findings, we relied on Lincoln and Guba (1985) criteria, namely credibility, transferability, confirmability, and dependability. Primarily, the researchers utilized peer review and member-checking to enhance the credibility of the findings. For peer review, the researchers met at multiple times, including when the study design was drafted and finalized, prior to data collection, after the completion of narrative frames, after each interview, following completion of data collection, during initial coding and themes development, and when reaching agreement on the final themes. During these meetings, consensus among the three researchers was required to advance through the different stages of research. Moreover, member checking was used with participants at both stages of data collection. After participants completed the narrative frames electronically, the first two researchers reviewed the completed frames with each participant. To further explain this, participants were asked to confirm that the written frames represented their experiences accurately, allowing them the opportunity for modification before finalizing them. Furthermore, following transcription of interviews, the first two researchers were engaged with each participant in follow-up interactions, giving them freedom to exclude any information or add new insights. As part of this process, initial findings were also informally shared with participants, enabling them to review or clarify any of their recorded perceptions or ideas. In addition, by providing a thick and contextualized description of the participant's experiences and through utilizing researcher reflexivity during our meetings, we intended to enhance both transferability and confirmability of the findings. Dependability, on the other hand, was maintained through providing a detailed methodological account of the research process.

### 3.4 Researchers' positionality

As both educators and researchers, our positionality is influenced by our growing interest in the use of AI, especially in academic writing, where it has been an increasingly discussed phenomenon in higher education. At the micro level, our interest lies at the intersection of the growing integration of AI tools in learning environments and teachers' conflicting responses to them. In such a context, we believe that any emerging (or lack of) institutional AI policies act as an influential factor that regulates AI usage at the macro level. In addition, we acknowledge that our prior and current experiences with academic writing and technology within higher educational contexts may influence our interpretation of teachers' responses with regard to AI-related practices and policy implementation. While we consider the promising benefits of AI in teaching and learning, we are also aware of teachers' growing concerns about issues related to academic integrity, authorship, and institutional accountability. Nevertheless, we have been continuously engaged in a reflective practice throughout the stages of this research to ensure the exclusion of any potential biases. This was done through regular meetings in which we were involved in constant member checking, in which we openly shared our views and utilized mutual peer feedback.

## 4 Results

The following section presents more details regarding how the participants viewed and navigated AI policies through themes that emerged from their reflections on their institution's plans regarding the emergence of AI. These themes revolve around the lack of clear institutional guidance, the sense that institutional approaches to AI are largely top-down, and the perception that AI adoption is an unavoidable development within higher education.

### 4.1 Absence or ambiguity of institutional AI policies

One of the themes that predominated the interviews was the perception that institutional policies of AI are either absent or inexplicitly defined. Some teachers (T3, T4, T5, T6, and T7) frequently pointed out the absence of clear institutional communication regarding how AI should be addressed in both teaching and assessment. Their responses often focused on the lack of written guidance as well as the uncertainty that this lack of clarity created in classroom practice. To begin with, teachers mentioned that, regardless of the increasing presence of AI tools in academic settings, their institution has yet to provide written guidance that explicates acceptable and unacceptable uses of these technologies. This lack of clarity left teachers to navigate AI-related challenges on their own. For example, T4 stated, “there's no proper guidance in terms of what we can do, what we can say no to. It's not very clear still.” In support of this, T3 also wrote:

The institution's expectations on AI were unclear. Initially, I felt unsure about the use of AI because comments were vague, conflicting and given in an “open discussion” environment…. However, these expectations are now still ongoing and in the process of being clarified (Narrative frame).

Similarly, T6 also highlighted the absence of a formal institutional framework governing AI use:

As far as I know, we haven't received a written institutional policy from the administration…. From the institutional viewpoint, it should define what AI is, how the institution is going to be using it, or if it's not going to be using it. What are the acceptable uses of AI? What are the unacceptable uses of AI? It should have a board, right? (Interview).

Another teacher expressed surprise that clearer policy frameworks had not yet been developed, especially given the time that has passed since AI tools became widely accessible. T7 stated:

“I expected some clear directions and clear policies to have come out by now. I think the universities had more than enough time to formulate some clear policies and the fact that it still remains unclear to me” (Interview).

On another note, T5 reflected on the challenges of evaluating the work of students who used AI irresponsibly, amidst the absence of clear institutional direction:

“So it's not actually the frustration, it's not even so much the frustration with the students. It's the frustration that if this unoriginal work is being accepted as original work.” T3 also shared:

One memorable event with regards to my institution's stance on AI was when I was asked to grade something I knew to be produced using AI. This experience made me think my profession had been devalued (Narrative frame).

Without explicit guidelines, teachers were often required to interpret institutional expectations on their own. This lack of guidance and ambiguity led teachers to uncertainty and frustration, as well as some inconsistencies in how AI use is addressed across classrooms.

### 4.2 Top-down institutional approaches to AI adoption

Alongside participants frequently expressing concerns about the lack of clear policy direction, another theme emerged that reflected how institutional approaches to AI adoption were largely designed and implemented through a top-down approach. Teachers repeatedly referred to institutional decision-making as being shaped at the administrative level, while teachers were excluded from the decision-making process and were consequently and merely left to implement such decisions. While participants did acknowledge the fact that universities may feel compelled to address AI due to its growing presence, some teachers referred to a gap between institutional priorities and the realities of teaching. These accounts reflected how participants positioned themselves in relation to institutional decisions. T2 stated:

These expectations are now shifting toward a more constructive, “AI-literate” approach where the focus is on ethical usage rather than total ban. The current institutional policies on AI are geared toward integration rather than elimination (Narrative Frame).

T2 also reflected on how openness to AI has started to emerge on the macro level:

One memorable event with regards to my institution's stance on AI was when our department held its first workshop on using AI to generate language-learning materials, showing a sudden openness to the technology (Narrative frame).

While T4 also showed his concerns, he found that the institution was compelled to eventually accept AI, subsequently looking for solutions to AI-challenges at the administration-level:

I don't like it. I'm not comfortable. I don't think it's the best thing to do. But I think maybe it's understandable from the university's perspective because they realize it's something that is there and other universities are using it (Interview).

T2 supported this further by illuminating how his views on AI differed from the institution's focus:

Compared to my own standpoint on AI, my institution's vision feels slightly more cautious, as I am eager to tighten or restrict AI use by students, while the administration remains focused on data security (Narrative frame).

On another note, T5 emphasized the importance of involving experienced educators in the development of AI policies:

I also think that the university, while I'm an employee here, that the university actually needs to look at the profile of some of the teachers that they have… who actually has the experience and who has the depth of knowledge and real understanding to help create some of those policies and make them a little more realistic (Interview).

Overall, teachers expressed their concern that institutional AI adoption was shaped largely at the administrative level, with limited teacher involvement.

### 4.3 Perception of AI adoption as an unavoidable development

In addition to participants' concerns regarding institutional approaches to AI, another theme emerged that indicated that the adoption of AI within universities is ultimately unavoidable. Whether they perceived it as an opportunity or challenge, participants repeatedly referred to AI as something that is already present within academic contexts and difficult to resist. This belief is clearly reflective of how AI is becoming an essential part of teaching and learning environments, which also needs to be considered in policy design. T1 stated:

Overall, I would describe my institution's expectations toward AI integration as enlightening because it is the future regardless of whether you disagree or not. AI will play a strategic part in our lives and education (Narrative frame).

In addition, a number of teachers described a sense of acquiescence to the growing presence of AI in educational settings. T4 reflected this sense of inevitability:

Well, while I'm not accepting [AI] emotionally, but I sort of gave up. You know, you just have to give up and say, yeah, he is where he is. You can do nothing about it, especially within the context of work (Interview).

A similar thought was expressed by T9, who likewise viewed institutional adoption of AI as predictable: “I think they will adopt it. I think this is inevitable. They cannot just ignore it. I think they're ready for it, and they're looking for it.”

In a similar vein, T10 echoed this perspective while accounting for the practical pressures associated with AI use: “In the beginning, as far as I remember in the beginning, it was not allowed to use AI in Saudi Arabia in our institution especially. But you, I mean, you cannot survive without it.” While there is indeed a growing pressure on teachers to utilize AI in classrooms, AI in parallel is evolving rapidly. T1 also maintained that current institutional policies on AI are designed so that students can use them effectively: “students can benefit from the use of AI in many ways, and it can enhance the creativity of learning but of course AI must be monitored” (Narrative frame). Overall, teachers described AI as increasingly integrated into academic practice, making its avoidance no longer feasible.

## 5 Discussion

This study set out to explore the perspectives of writing teachers on the policies related to AI within their institution. The most prominent finding to emerge from the data is that the perceived inevitability and pervasive presence of AI for writing teachers underscore an urgent need for structured, context-appropriate institutional policies. According to the participants' narratives, a structured and context-appropriate AI policy could account for different scenarios, such as what to do when students submit work they completed using AI irresponsibly. A further insight into the details of such policies indicates that the ambiguity of policies could place a significant pedagogical burden on teachers, leading to a possible disruption of the learning process. As declared by the participants, the ill-defined policies attracted uncertainty in the classroom, as teachers were not always able to determine the limits of what is acceptable in regard to AI usage by students and/or teachers and what is not. What also lies at the heart of this discussion is the peripheral role given to teachers in the making of those policies. To teachers, failing to meaningfully include them in the crafting phase of the policies may result in mandates that are difficult to apply to classroom realities, thereby contributing to a cycle of policy ambiguity and uncertainty.

In line with previous research, these findings indicate that institutional responses to AI were mainly characterized by uncertainty, inevitability, and unresolved tensions between adaptation and pedagogical practice. Although institutions increasingly tend to frame AI as inevitable and strategically necessary (Zawacki-Richter et al., 2019), participants stated that they have been left to negotiate its ethical and instructional implications independently. This was reflective of broader arguments that educational technologies are often institutionally promoted before their pedagogical consequences are fully understood. In some cases, this institutional positioning was also recognized as different from that of the participants. Some narratives illustrated a tension between the participant's personal reservations and their understanding of the institutional point of view. While such comments expressed discomfort with AI, they acknowledged that universities may feel compelled to address it simply because it already exists and is increasingly present within higher education. In this sense, participants recognized that institutional attention toward AI may stem less from enthusiasm for the technology itself and more from an acknowledgment that it has become a reality that universities cannot easily ignore (Williamson and Eynon, 2020). This distinction is important because it suggests that teachers' ambivalence should not necessarily be interpreted as resistance to AI itself. Rather, their concerns appear to be shaped by the conditions under which AI is introduced, governed, and translated into classroom practice.

Furthermore, participants' accounts of unclear guidance and inconsistent expectations reinforced earlier observations that institutional responses toward AI have largely been fragmented (Cotton et al., 2024; Korseberg and Elken, 2025; Spivakovsky et al., 2023). The emphasis on written policy appeared to be rather deliberate and suggested that informal conversations or uncertain recommendations may have been present within departments, yet were not perceived as authoritative enough to support teachers in their classroom decision-making. Thus, the concern was not simply whether institutional attention to AI existed, but whether the formal policy and related guidance were sufficiently clear and applicable to support teachers in making classroom decisions. At the same time, the findings also problematize recent claims that institutions are moving toward coherent frameworks centered on AI literacy and ethical integration (Jin et al., 2025; McDonald et al., 2025). Although such discourse may exist institutionally, participants' experiences in this study suggested that these frameworks had not yet been translated into clear classroom practice, thereby leaving the responsibility for managing AI on teachers without proper institutional support. In this sense, the findings point to a gap between institutional governance and policy enactment where the existence of a formal policy does not necessarily ensure that its expectations and/or applications are sufficiently clear or readily translated into classroom practice.

The study also concluded that the ambivalence toward AI was not necessarily shaped by resistance to technology itself, but rather by worries over diminishing pedagogical authority. Similar to previous studies (e.g., Bearman et al., 2023; Mamo et al., 2024; Petricini et al., 2024), participants acknowledged AI's inevitability while remaining cautious about its educational implications. In some cases, the frustration expressed by participants was not directed at students themselves but at the broader institutional environment in which AI-generated work may be undetected or accepted as original. This concern highlights the tension between expectations of originality in academic writing and the growing presence of AI tools that can produce text on behalf of students (Barrett and Pack, 2023; Gustilo et al., 2024). Further, teachers' experiences also highlighted how top-down institutional approaches may intensify such concerns by limiting faculty involvement in policy formation. Participants noted that institutional policymaking should draw more directly on the expertise of teachers. This was evident in participants' emphasis on words such as “experience” and “depth of knowledge,” which suggests that teachers think of themselves as valuable contributors to policy formation discussions, especially when those policies directly affect classroom practices (Beck, 2024). Taken together, these findings suggest that teacher agency is shaped not only by teachers' individual attitudes toward AI, but also by the extent to which they are positioned as participants in institutional decision-making. When teachers are expected to enact AI-related expectations without meaningful involvement in their development, professional autonomy may become constrained by the need to interpret and apply institutional expectations at the classroom level. Thus, the complication of the issue identified in this study is not simply whether teachers accept or reject AI, but how institutional governance shapes the conditions under which teachers exercise their professional judgment.

This perspective contributes to existing scholarship by highlighting the relationship between institutional AI governance and teachers' professional agency in writing instruction. While previous research has highlighted fragmented institutional responses to AI and teachers' concerns about its pedagogical implications (Cotton et al., 2024; Korseberg and Elken, 2025; Spivakovsky et al., 2023), the present findings further suggest that these concerns may also be connected to how responsibility for managing AI is distributed between institutional decision-makers and teachers. In particular, the findings show how unclear institutional expectations can shift much of the practical responsibility for interpreting and managing AI toward individual teachers, creating tensions between top-down institutional expectations and the realities of classroom practice. The contribution of the study therefore lies not in presenting AI as a new source of teacher resistance, but in illustrating how the governance of AI can become a condition through which teachers negotiate their pedagogical authority and professional autonomy.

The findings of this study point to vital practical and research implications. First, for AI-related institutional policies to be effective, they must offer explicit, unambiguous operational definitions of acceptable and unacceptable AI use. This clarity is critical for language teachers who require concrete benchmarks to navigate classroom uncertainties. Second, to ensure these policies are realistic and contextually appropriate, institutions must involve relevant teachers in the policy-drafting phase, such as writing teachers, to develop AI-related policies for writing courses. Given their direct engagement with student practices, teachers' insights are invaluable for bridging the gap between top-down administrative expectations and bottom-up classroom realities. These implications are particularly relevant to the present context, where teachers' accounts suggest that a formal institutional policy does not, by itself, eliminate uncertainty at the classroom level. As for further research investigations, aiming to collect data from different entities within these institutions, such as policymakers, admin-level employees, and language teachers, other than writing, is one way to frame this issue more holistically. Students' voices are also important for further exploration and analysis.

The Saudi context is relevant to interpreting these findings in two related ways. First, participants' perception of AI as an increasingly inevitable institutional reality can be situated within Saudi Arabia's broader national orientation toward AI adoption and digital transformation. Through initiatives associated with Vision 2030 and the National Strategy for Data and AI, AI has been positioned as a strategic national priority across multiple sectors, including education (Saudi Data and Artificial Intelligence Authority, 2020). This broader orientation provides an important context for understanding why participants perceived AI as difficult for higher education institutions to ignore, even as questions surrounding its pedagogical implementation remained unresolved. Second, the findings should be understood in relation to the prominence of English-medium instruction in Saudi higher education (Aljehani and Modiano, 2024). For writing teachers working in this context, generative AI directly intersects with the teaching and assessment of academic writing, raising particular concerns surrounding originality, authorship, and students' independent language production. These contextual factors do not suggest that the findings are representative of all Saudi higher education institutions; rather, they help situate the participants' experiences within a broader national and educational environment in which both AI adoption and English-mediated academic practices hold increasing importance.

In light of the findings of this study, a few limitations must be acknowledged. One limitation includes the inability to generalize the findings to other contexts, as the data in this case study were collected from one university in Saudi Arabia. However, the detailed description of the research context and the participants' accounts was provided to allow readers to assess the potential transferability of the findings to similar contexts (Lincoln and Guba, 1985). Secondly, since most writing teachers in the context of this study were expatriate, the study lacks insights from Saudi teachers. This was due to the fact that most expatriate teachers were assigned to teach writing courses within the setting of this study. Further research on Saudi writing teachers' responses to AI policies is needed to understand how their views might agree or differ with the results of this study. Finally, as AI is evolving rapidly, it is crucial to understand policy formation and implementation over time, given that teachers' views in this study represent their experiences within a specific timeframe and might change over time and experience. Longitudinal and large-scale studies might offer more insights into understanding how AI policies are implemented and maintained through consistent teaching practices.

## 6 Conclusion

This study explored how writing teachers viewed and navigated AI institutional policies within one university in Saudi Arabia. Following a qualitative approach, the findings revealed that the teachers recognized AI policies as either absent or ambiguous, leading them often to interpret these policies on their own. The findings also highlighted how institutional AI policies followed a top-down approach, where teachers were left only to direct implementation. However, the teachers underlined the importance of AI within today's world of higher education, stressing that it is an unavoidable development that will play an essential part in their future teaching practices. While it is important to include such teachers in the policy design and implementation phases, the study also suggests that institutional AI policies are actively interpreted and negotiated by teachers. These findings underscore the importance of developing AI policies that are clear, flexible, and responsive to the realities of writing instruction. Future research on how teachers' pedagogical beliefs and professional experiences with AI evolve over time is needed to understand how AI policies could translate into pedagogically meaningful and practically applicable teaching practices.

## Data Availability

The datasets presented in this article are not readily available because they compromise the privacy of the participants. Requests to access the datasets should be directed to farisabdulrahman4@gmail.com.
